# Four-year trends in health findings among hospital staff an ecological time-series study 2022–2025

**DOI:** 10.3389/fpubh.2026.1890585

**Published:** 2026-10-07

**Authors:** Dan Zhang, Rong Xing, Yuanling Wu, Junyan Zhang, Zhigan Lv, Fang Wang, Ruiying Wang, Fang Gao

**Affiliations:** 1 Department of Human Resources, Shanxi Bethune Hospital, Shanxi Academy of Medical Sciences, Tongji Shanxi Hospital, Third Hospital of Shanxi Medical University, Taiyuan, China; 2 Department of Preventive Healthcare, Shanxi Bethune Hospital, Shanxi Academy of Medical Sciences, Tongji Shanxi Hospital, Third Hospital of Shanxi Medical University, Taiyuan, China; 3 Department of Clinical Laboratory, Shanxi Bethune Hospital, Shanxi Academy of Medical Sciences, Tongji Shanxi Hospital, Third Hospital of Shanxi Medical University, Taiyuan, China; 4 Department of Clinical Epidemiology and Evidence-Based Medicine, Shanxi Bethune Hospital, Shanxi Academy of Medical Sciences, Tongji Shanxi Hospital, Third Hospital of Shanxi Medical University, Taiyuan, China; 5 Health Management Center, Shanxi Bethune Hospital, Shanxi Academy of Medical Sciences, Tongji Shanxi Hospital, Third Hospital of Shanxi Medical University, Taiyuan, China; 6 Department of Pulmonary and Critical Care Medicine, Shanxi Bethune Hospital, Shanxi Academy of Medical Sciences, Tongji Shanxi Hospital, Third Hospital of Shanxi Medical University, Taiyuan, China

**Keywords:** health examination, hospital staff, occupational health, sex differences, temporal trends

## Abstract

**Background:**

Annual health examinations provide an opportunity to monitor major health findings among hospital staff. This study described examination uptake, sex-specific differences, and four-year trends in major health findings using institutional annual health examination reports.

**Methods:**

An ecological time-series study was conducted using aggregated annual health examination reports from 2022 to 2025. Examination uptake, age composition, sex-specific differences, and temporal trends in major health findings were evaluated using annual institutional summary data. Annual sex differences were assessed using chi-square or Fisher’s exact tests. Temporal trends across the four examination cycles were evaluated using the Cochran-Armitage trend test.

**Results:**

A total of 14,077 annual examination records were included. Examination uptake remained above 90% across all four examination cycles, with female staff consistently showing higher uptake than male staff (all *p* < 0.001). Dental and Periodontal Diseases remained the most frequently detected health finding, whereas Dyslipidemia, Thyroid Nodules, and Overweight or Obesity were also commonly detected. Significant temporal changes were observed across all major health findings (all *p* ≤ 0.02). Marked sex-specific differences were identified, with metabolic abnormalities more common in male staff and thyroid-related abnormalities more common in female staff. The prevalence of Breast Nodules among examined female staff increased significantly across the four examination cycles (*p* < 0.001).

**Conclusion:**

Major health findings remained common among hospital staff over the four-year study period, with significant temporal changes and clear sex-specific differences. These findings support targeted health monitoring and preventive management in hospital settings.

## Introduction

Healthcare workers are a critical occupational population for chronic disease prevention because their work is often characterized by shift schedules, high cognitive and emotional demands, irregular meals, sleep disruption, and limited time for physical activity. These exposures do not affect all staff members equally, but they may contribute to clustering of cardiometabolic, endocrine, and lifestyle-related abnormalities. A recent systematic review and meta-analysis linked shift work with a higher risk of metabolic syndrome, with sleep, sex, and shift pattern acting as important modifiers (1). In parallel, China has experienced a substantial burden of metabolic risk factors, including Dyslipidemia, Overweight or Obesity, Hepatic Steatosis, and Hyperuricemia (2–5). Regular health examinations in hospital employees therefore provide an opportunity to identify early abnormalities, monitor temporal changes, and design targeted workplace health management programs.

Routine staff health examinations are particularly valuable when they combine laboratory testing, anthropometric assessment, ultrasonography, oral examination, and sex-specific screening. These metabolic abnormalities are clinically important because they are modifiable and frequently cluster within individuals, thereby amplifying cumulative cardiometabolic risk and increasing the likelihood of long-term adverse outcomes (6–8). Thyroid Nodules and autoimmune thyroiditis are also frequently detected in health examination settings, especially with widespread thyroid ultrasonography, and both Chinese health examination data and global meta-analytic evidence suggest that Thyroid Nodules are common in adult screening populations (9, 10). Breast Nodules, although often benign, require standardized imaging classification and follow-up pathways to identify higher-risk lesions, particularly because breast cancer remains one of the most common cancers among women worldwide (11). Dental and periodontal diseases are also important because they are highly prevalent, affect quality of life, and contribute substantially to the global burden of oral disease (12).

Despite the widespread use of annual health examinations in China, institution-level longitudinal summaries of hospital employees remain limited, particularly in terms of scientific analysis of multi-year and sex-specific patterns. Such data can help occupational health teams move from passive screening to active prevention, particularly when abnormalities show consistent upward trends or marked sex disparities.

To address this gap, this study provides a comprehensive assessment of multi-year and sex-specific patterns in health examination abnormalities among hospital employees, covering metabolic, endocrine, oral, and female-specific screening domains.

## Methods

### Study design and data source

This study used aggregated annual staff health examination reports from Shanxi Bethune Hospital between 2022 and 2025 to describe examination uptake, sex-specific differences, and temporal trends in major health examination abnormalities. Eight common abnormalities with consistent annual data were included, including Breast Nodules, which were assessed only among female employees as part of sex-specific screening, Dental and Periodontal Diseases, Dyslipidemia, Hepatic Steatosis, Hashimoto Thyroiditis, Hyperuricemia, Overweight or Obesity, and Thyroid Nodules. The objective was to characterize the burden and trajectory of these findings and to provide evidence for targeted workplace health management among hospital employees.

This was an ecological time-series analysis of annual employee health examination data from Shanxi Bethune Hospital, Shanxi, China, covering four examination cycles from 2022 to 2025. The analysis followed the general reporting principles of the STROBE statement for observational studies (13). Data were extracted from annual group health examination reports generated by the Health Management Center and from a structured summary table of examination uptake, abnormality counts, prevalence estimates, sex-specific comparisons, and temporal trend tests.

The annual examination periods were January 10 to May 17, 2022; January 18 to October 17, 2023; January 1 to November 12, 2024; and January 1 to December 3, 2025. The source reports described the health examination program as an institutional employee health assessment intended to summarize group health status and support health education and intervention. The present analysis used only aggregated, de-identified annual counts. No individual identifiers were available in the analytic dataset.

### Participants

The target population comprised all employees scheduled to participate in the annual staff health examination program at Shanxi Bethune Hospital between 2022 and 2025. Annual institutional health examination reports provided aggregated information on the expected number of examinees, actual examined number, sex distribution, age distribution, and detected health examination abnormalities. Examination uptake was defined as the proportion of employees who completed the examination among those expected to attend. Because only annual aggregated data stratified by year and sex were available, the unit of analysis was the annual examination record rather than a unique individual. Accordingly, this study was designed as an ecological time-series surveillance rather than individual longitudinal follow-up.

### Variables and outcomes

The main descriptive variables included calendar year, sex, expected number of examinees, examined number of employees, examination uptake rate, and age distribution. Age information was summarized in the annual reports as mean age and categorical age distribution (<45, 45 to 54, 55 to 64, and ≥65 years), consistent across all study years. The primary outcomes were annual changes in the prevalence of major detected health examination abnormalities, including Dental and Periodontal Diseases, Dyslipidemia, Hepatic Steatosis, Hashimoto Thyroiditis, Hyperuricemia, Overweight or Obesity, Thyroid Nodules, and Breast Nodules among examined female employees. Secondary descriptive outcomes included annual examination uptake and changes in age-group composition. Total cholesterol (TC) was measured using the enzymatic method. Low-density lipoprotein cholesterol (LDL-C) and high-density lipoprotein cholesterol (HDL-C) were measured using direct assays. Triglycerides (TG) were measured using the glycerol phosphate oxidase-peroxidase method. Uric acid was measured using the uricase-peroxidase method. Thyroid-stimulating hormone (TSH) and free thyroxine (FT4) were measured using chemiluminescent immunoassays. All reagents were supplied by Beckman Coulter, Inc. (USA). Breast nodules, thyroid nodules, and abdominal ultrasonographic examinations were performed using ACUSON Sequoia Silver and ACUSON Sequoia i ultrasound systems (Siemens Shanghai Medical Equipment Ltd., Shanghai, China), as well as the LOGIQ E10s ultrasound system (GE Healthcare Ultrasound, Korea).

The criteria of the examination abnormalities are

1. Dental and periodontal diseases:
  Dental diseases were identified based on the presence of cavitation, enamel or dentin involvement, and lesion characteristics involving smooth surfaces, proximal surfaces, or pit-and-fissure areas, with severity graded according to the International Caries Classification and Management System (ICCMS) and the International Caries Detection and Assessment System (ICDAS) (14).
  Periodontal diseases were assessed based on visual examination of gingival color, gingival contour, gingival consistency, gingival surface texture, and dental plaque/calculus accumulation, together with periodontal probing indicators including bleeding on probing (BOP), probing pocket depth (PPD), clinical attachment loss (CAL), furcation involvement, gingival recession, tooth mobility, and tooth displacement (15).
2. Dyslipidemia:
  Abnormality of TC > 200 mg/dL (5.18 mmol/L) (16)
  Abnormality of LDL-C > 160 mg/dL (4.14 mmol/L) (16)
  Abnormality of TG > 150 mg/dL (1.7 mmol/L) (17)
  Abnormality of HDL-C < 40 mg/dL (1.04 mmol/L) (16)
3. Hepatic steatosis was assessed by abdominal ultrasonography based on diffuse increased hepatic echogenicity (bright liver), increased hepatorenal echo contrast, deep attenuation, and blurring of the portal vein and intrahepatic vessel walls (18).
4. Hashimoto thyroiditis: elevated TSH levels (>5 mIU/L) with normal or reduced FT4 levels (<58 nmol/L), together with characteristic thyroid ultrasonographic findings (19, 20). Thyroid autoantibodies were not routinely assessed because this was an employee health examination program.
5. Hyperuricemia was defined as a serum uric acid level >420 μmol/L (approximately 7.0 mg/dL) in males and >360 μmol/L (approximately 6.0 mg/dL) in females (21).
6. Overweight or Obesity: BMI ≥ 24 kg/m^2^ for overweight and BMI ≥ 28 kg/m^2^ for obesity (22).
7. Thyroid nodules and breast nodules were identified based on ultrasonographic findings (23, 24)

### Statistical analysis

Counts and percentages were used to summarize examination uptake and abnormality prevalence. Overall prevalence for each abnormality was calculated as the number of detected cases divided by the total number of examined employees in the corresponding year. Sex-specific prevalence was calculated as the number of detected cases in men or women divided by the number of examined men or women in the corresponding year. Sex differences in annual prevalence were assessed using chi-square tests or Fisher’s exact tests, as appropriate. Linear trends across years were assessed using the Cochran-Armitage trend test. A two-sided *p* value <0.05 was considered statistically significant. Because only aggregated data were available, multivariable adjustment for age, department, occupation, shift work, medication use, or repeated participation by the same individuals was not possible.

## Results

### Examination uptake and participant profile

Examination uptake remained consistently high throughout the study period, exceeding 90% in all four annual examination cycles, with an overall uptake rate of 93.53%. Female employees consistently demonstrated higher examination uptake than male employees, and sex differences remained statistically significant in each study year (all *p* < 0.001). No significant linear change in examination uptake was observed over time in the overall population, male employees, or female employees (all *p* > 0.05; Table 1).

**Table 1:** Examination uptake rate.

Year	All employees			Male			Female			χ^2^	*p*-value
	Expected	Examined	Uptake rate	Expected	Examined	Uptake rate	Expected	Examined	Uptake rate		
2022	3,431	3,251	94.75%	814	735	90.29%	2,617	2,516	96.14%	42.68	<0.001
2023	3,797	3,527	92.89%	947	816	86.17%	2,850	2,711	95.12%	86.32	<0.001
2024	3,913	3,615	92.38%	970	833	85.88%	2,943	2,782	94.53%	77.64	<0.001
2025	3,909	3,684	94.24%	961	849	88.35%	2,948	2,835	96.17%	81.73	<0.001
z- value			1.76			0.68			1.87		
*p*-value			0.08			0.50			0.06		

Temporal trends across years were evaluated using the Cochran-Armitage trend test, and the reported z-values represent the corresponding test statistics.

The examined workforce was predominantly young to middle-aged across all study years. The mean age increased modestly from 33 years in 2022 to 35 years in 2025, including an increase from 35 to 38 years among male employees and from 32 to 34 years among female employees. Employees younger than 45 years consistently accounted for approximately 90% of examined employees throughout the study period, although the proportion of employees aged 45 years or older increased slightly over time (Table 2).

**Table 2:** Yearly age composition of examined male employees, 2022–2025.

Sex	Category	Year 2022	Year 2023	Year 2024	Year 2025
Male	Total number of examined employees	735	816	833	848
	Number of age below 45 (proportion)	645 (87.76%)	667(81.74%)	682 (81.87%)	695 (81.96%)
	Number of age 45 ~ 54 (proportion)	68 (9.25%)	92 (11.27%)	96 (11.52%)	92 (10.85%)
	Number of age 55 ~ 64 (proportion)	19 (2.59%)	35 (4.29%)	32 (3.84%)	39 (4.60%)
	Number of age ≥65 (proportion)	3 (0.41%)	22 (2.70%)	23 (2.76%)	22 (2.59%)
Female	Total number of examined employees	2,516	2,711	2,782	2,836
	Number of age below 45 (proportion)	2,373 (94.32%)	2,502 (92.29%)	2,572 (92.45%)	2,614 (92.17%)
	Number of age 45 ~ 54 (proportion)	119 (4.73%)	153 (5.64%)	151 (5.43%)	157 (5.54%)
	Number of age 55 ~ 64 (proportion)	17 (0.68%)	34 (1.25%)	41 (1.47%)	46 (1.62%)
	Number of age ≥65 (proportion)	7 (0.28%)	22 (0.81%)	18 (0.65%)	19 (0.67%)

### Overall burden and temporal patterns of major abnormalities

Dental and Periodontal Diseases represented the most frequently detected abnormality throughout the study period, with annual prevalence ranging from 65.98 to 75.76% (Table 3). Dyslipidemia and Hepatic Steatosis were detected in 34.54 to 41.63% and 18.18 to 24.54% of examined employees, respectively (Tables 4, 5). Hashimoto Thyroiditis and Hyperuricemia showed annual prevalence ranging from 13.66 to 17.05% and 9.81 to 15.30%, respectively (Tables 6, 7). Overweight or Obesity and Thyroid Nodules were detected in 29.47 to 33.52% and 32.17 to 42.48% of examined employees, respectively (Tables 8, 9; Figures 1A,B). Trend analyses demonstrated significant temporal changes across all seven abnormalities (all *p* ≤ 0.02).

**Table 3:** Prevalence of detected dental and periodontal diseases in examined employees.

Year	Examined employees			Examined male			Examined female			χ^2^	*p*-value
	Number	DPD	Prevalence	Number	DPD	Prevalence	Number	DPD	Prevalence		
2022	3,251	2,463	75.76%	735	521	70.88%	2,516	1942	77.19%	12.30	<0.001
2023	3,527	2,327	65.98%	816	530	64.95%	2,711	1797	66.29%	0.50	0.481
2024	3,615	2,522	69.76%	833	561	67.35%	2,782	1961	70.49%	3.00	0.083
2025	3,684	2,646	71.82%	849	611	71.97%	2,835	2035	71.78%	0.01	0.916
z- value			3.24			3.26			2.31		
*p*-value			0.001			0.001			0.02		

Temporal trends across years were evaluated using the Cochran-Armitage trend test, and the reported z-values represent the corresponding test statistics. DPD, dental and periodontal diseases.

**Table 4:** Prevalence of detected dyslipidemia in examined employees.

Year	Examined employees			Examined male			Examined female			χ^2^	*p*-value
	Number	DLP	Prevalence	Number	DLP	Prevalence	Number	DLP	Prevalence		
2022	3,251	1,123	34.54%	735	441	60.00%	2,516	682	27.11%	272.20	<0.001
2023	3,527	1,376	39.01%	816	526	64.46%	2,711	850	31.35%	288.94	<0.001
2024	3,615	1,505	41.63%	833	568	68.19%	2,782	937	33.68%	314.12	<0.001
2025	3,684	1,404	38.11%	849	563	66.31%	2,835	841	29.66%	372.04	<0.001
z- value			6.55			4.79			6.02		
*p*-value			<0.001			<0.001			<0.001		

Temporal trends across years were evaluated using the Cochran-Armitage trend test, and the reported z-values represent the corresponding test statistics. DLP, Dyslipidemia.

**Table 5:** Prevalence of detected hepatic steatosis in examined employees.

Year	Examined employees			Examined male			Examined female			χ^2^	*p*-value
	Number	HS	Prevalence	Number	HS	Prevalence	Number	HS	Prevalence		
2022	3,251	591	18.18%	735	329	44.76%	2,516	262	10.41%	451.19	<0.001
2023	3,527	748	21.21%	816	408	50.00%	2,711	340	12.54%	526.67	<0.001
2024	3,615	819	22.66%	833	428	51.38%	2,782	391	14.05%	509.69	<0.001
2025	3,684	904	24.54%	849	448	52.77%	2,835	456	16.08%	474.79	<0.001
z- value			7.25			5.01			6.85		
p-value			<0.001			<0.001			<0.001		

Temporal trends across years were evaluated using the Cochran-Armitage trend test, and the reported z-values represent the corresponding test statistics. HS, Hepatic steatosis.

**Table 6:** Prevalence of detected Hashimoto thyroiditis in examined employees.

Year	Examined employees			Examined male			Examined female			χ^2^	*p*-value
	Number	HT	Prevalence	Number	HT	Prevalence	Number	HT	Prevalence		
2022	3,251	444	13.66%	735	56	7.62%	2,516	388	15.42%	29.37	<0.001
2023	3,527	527	14.94%	816	71	8.70%	2,711	456	16.82%	32.53	<0.001
2024	3,615	548	15.16%	833	70	8.40%	2,782	478	17.18%	38.41	<0.001
2025	3,684	628	17.05%	849	87	10.25%	2,835	541	19.08%	36.06	<0.001
z- value			6.41			4.18			6.30		
*p*-value			<0.001			<0.001			<0.001		

Temporal trends across years were evaluated using the Cochran-Armitage trend test, and the reported z-values represent the corresponding test statistics. HT, Hashimoto Thyroiditis.

**Table 7:** Prevalence of detected hyperuricemia in examined employees.

Year	Examined employees			Examined male			Examined female			χ^2^	*p*-value
	Number	HUA	Prevalence	Number	HUA	Prevalence	Number	HUA	Prevalence		
2022	3,251	319	9.81%	735	175	23.81%	2,516	144	5.72%	210.26	<0.001
2023	3,527	477	13.52%	816	226	27.70%	2,711	251	9.26%	182.31	<0.001
2024	3,615	553	15.30%	833	264	31.69%	2,782	289	10.39%	224.55	<0.001
2025	3,684	419	11.37%	849	224	26.38%	2,835	195	6.88%	246.61	<0.001
z- value			6.02			4.15			5.77		
*p*-value			<0.001			<0.001			<0.001		

Temporal trends across years were evaluated using the Cochran-Armitage trend test, and the reported z-values represent the corresponding test statistics. HUA, Hyperuricemia.

**Table 8:** Prevalence of detected overweight or obesity in examined employees.

Year	Examined employees			Examined male			Examined female			χ^2^	*p*-value
	Number	OW/OB	Prevalence	Number	OW/OB	Prevalence	Number	OW/OB	Prevalence		
2022	3,251	958	29.47%	735	400	54.42%	2,516	558	22.18%	284.53	<0.001
2023	3,527	1,130	32.04%	816	452	55.39%	2,711	678	25.01%	265.91	<0.001
2024	3,615	1,136	31.42%	833	454	54.50%	2,782	682	24.51%	267.50	<0.001
2025	3,684	1,235	33.52%	849	492	57.95%	2,835	743	26.21%	862.24	<0.001
z- value			6.48			3.76			6.28		
*p*-value			<0.001			<0.001			<0.001		

Temporal trends across years were evaluated using the Cochran-Armitage trend test, and the reported z-values represent the corresponding test statistics. OW/OB, overweight or obesity.

**Table 9:** Prevalence of detected thyroid nodules in examined employees.

Year	Examined employees			Examined male			Examined female			χ^2^	*p*-value
	Number	TN	Prevalence	Number	TN	Prevalence	Number	TN	Prevalence		
2022	3,251	1,046	32.17%	735	192	26.12%	2,516	854	33.94%	15.94	<0.001
2023	3,527	1,367	38.76%	816	277	33.95%	2,711	1,090	40.21%	10.36	0.001
2024	3,615	1,438	39.78%	833	285	34.21%	2,782	1,153	41.45%	13.99	<0.001
2025	3,684	1,565	42.48%	849	316	37.22%	2,835	1,249	44.06%	12.50	<0.001
z- value			8.54			5.54			8.19		
*p*-value			<0.001			<0.001			<0.001		

Temporal trends across years were evaluated using the Cochran-Armitage trend test, and the reported z-values represent the corresponding test statistics. TN, thyroid nodules.

**Figure 1:**
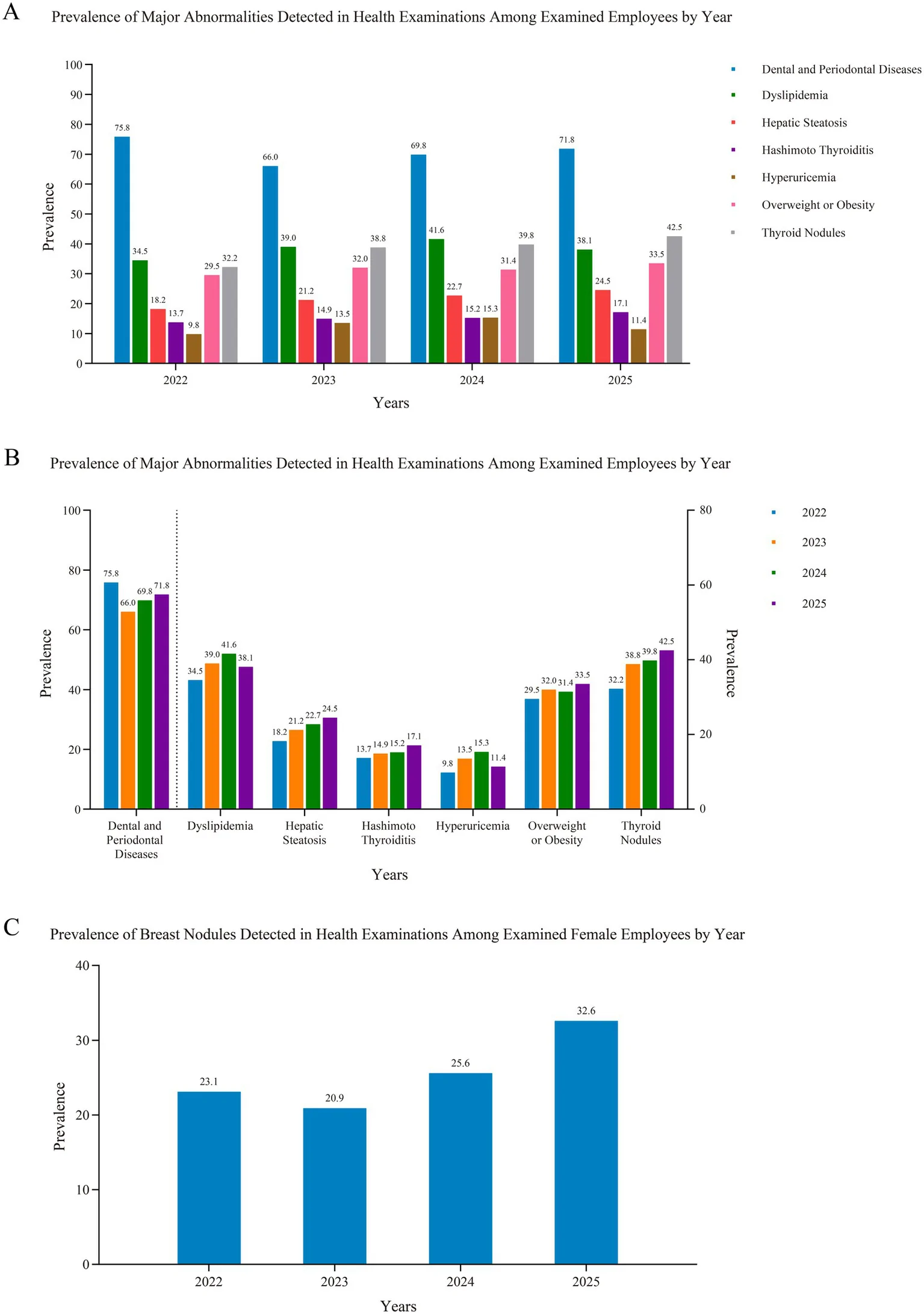
Annual prevalence of major detected health examination abnormalities among hospital employees from 2022 to 2025. **(A)** Annual prevalence of seven major detected health examination abnormalities among examined employees, including dental and periodontal diseases, dyslipidemia, hepatic steatosis, Hashimoto thyroiditis, hyperuricemia, overweight or obesity, and thyroid nodules. **(B)** Comparison of annual prevalence patterns for the same seven abnormalities across the four examination cycles. **(C)** Annual prevalence of breast nodules among examined female employees.

Breast Nodules were analyzed separately as a female-specific screening outcome. The annual prevalence of Breast Nodules among examined female employees was 23.05% in 2022, 20.91% in 2023, 25.59% in 2024, and 32.59% in 2025, with a significant upward temporal trend over time (*p* < 0.001; Table 10; Figure 1C).

**Table 10:** Prevalence of detected breast nodules among examined female employees.

Year	Examined employees			Examined male			Examined female			χ^2^	*p*-value
	Number	BN	Prevalence	Number	BN	Prevalence	Number	BN	Prevalence		
2022	—	—	—	—	—	—	2,516	580	23.05%	Fisher	<0.001
2023	—	—	—	—	—	—	2,711	567	20.91%	Fisher	<0.001
2024	—	—	—	—	—	—	2,782	712	25.59%	Fisher	<0.001
2025	—	—	—	—	—	—	2,835	924	32.59%	Fisher	<0.001
z- value									8.27		
*p*-value									<0.001		

Temporal trends across years were evaluated using the Cochran-Armitage trend test, and the reported z-values represent the corresponding test statistics. BN, breast nodules.

### Sex-specific differences

Marked sex-specific differences were observed across the major detected abnormalities. Dental and Periodontal Diseases were highly prevalent in both sexes throughout the study period, although a significant sex difference was observed only in 2022, with no significant between-sex differences in subsequent years. Male employees had substantially higher prevalence of Dyslipidemia, Hepatic Steatosis, Hyperuricemia, and Overweight or Obesity throughout the study period, whereas female employees showed consistently higher prevalence of Hashimoto Thyroiditis and Thyroid Nodules. Sex differences remained statistically significant for most abnormalities across all annual examination cycles (Tables 3–9).

## Discussion

This ecological time-series analysis of institutional health examination data from 2022 to 2025 revealed three major findings. First, metabolic abnormalities remained highly prevalent throughout the study period, particularly Dyslipidemia, Overweight or Obesity, Hepatic Steatosis, and Hyperuricemia. Second, marked sex disparities were consistently observed, with male employees showing substantially higher burdens of metabolic abnormalities, whereas thyroid-related abnormalities and Breast Nodules were predominantly identified in female employees. Third, several abnormalities, including Thyroid Nodules, Breast Nodules, Hepatic Steatosis, and Hashimoto Thyroiditis, demonstrated significant upward temporal trends, suggesting an expanding spectrum of health abnormalities identified through annual staff screening.

The sex-specific patterns observed in this study were clinically important. Male employees consistently showed substantially higher prevalence of Dyslipidemia, Hepatic Steatosis, Hyperuricemia, and Overweight or Obesity across all study years. The pooled male prevalence of Overweight or Obesity exceeded 55%, and nearly two-thirds of male examination records showed Dyslipidemia. These abnormalities likely reflect shared cardiometabolic pathways involving excess adiposity, insulin resistance, lifestyle behaviors, and circadian disruption, and are broadly consistent with metabolic risk patterns previously reported in Chinese adults and shift-working populations (1–5). The higher burden observed in male employees may additionally relate to sex-specific differences in visceral fat accumulation, alcohol consumption, dietary behaviors, and health-seeking patterns that have been consistently reported in occupational and population-based studies (25–28).

The increasing prevalence of Hepatic Steatosis is particularly noteworthy. Ultrasound-detected hepatic steatosis represents an important imaging manifestation of metabolic fatty liver disease, which has become a major public health concern globally and in China. Meta-analytic data suggest that the global prevalence of nonalcoholic fatty liver disease (NFLD), now termed metabolic dysfunction-associated steatotic liver disease (MASLD), is approximately 30% and continues to rise (4).

In this hospital employee population, Hepatic Steatosis increased from 18.18% in 2022 to 24.54% in 2025 and affected more than half of examined male employees in 2025. Although the present dataset cannot distinguish MASLD from alcohol-associated fatty liver or other causes, the strong co-occurrence of Overweight or Obesity and Dyslipidemia suggests that cardiometabolic prevention should be central to staff health management (2–4). These findings support the implementation of targeted workplace health education, metabolic risk awareness, and lifestyle intervention programs for employees with Hepatic Steatosis, particularly male staff members who consistently demonstrated the highest burden across all study years.

Thyroid and breast findings were more prominent among women. Thyroid Nodules increased steadily in both sexes and reached 42.48% overall in 2025, with a higher prevalence among women. This prevalence is broadly consistent with health examination cohorts and meta-analytic data using ultrasound, in which Thyroid Nodules are commonly detected and are more frequent in women and older adults (9, 10). The increase prevalence in Thyroid Nodules and Hashimoto Thyroiditis may reflect a combination of aging, improved ultrasound detection, true changes in thyroid disease burden, and possible autoimmune or environmental factors (10, 29–31).

Breast Nodules increased among women from 23.05% in 2022 to 32.59% in 2025. Most screen-detected Breast Nodules are benign, but high-risk breast imaging findings (BI-RADS ≥4) were consistently identified throughout the study period, increasing from four cases in 2022 to seven cases in both 2023 and 2024, and eight cases in 2025 (Supplementary Table S1). Beginning in 2024, institutional follow-up reports additionally documented two breast cancer cases annually, underscoring the importance of standardized breast imaging interpretation and closed-loop follow-up management in the context of the continuing global burden of breast cancer (11).

Dental and Periodontal Diseases were the most prevalent findings throughout the study period. Although the prevalence decreased after 2022 and then partially rebounded, it remained above 65% in every year. Oral disease is often underprioritized in occupational health programs, but its high frequency in this workforce is consistent with the substantial global burden of periodontal disease and suggests that oral health education, regular dental cleaning, early treatment of caries and periodontal disease, and improved access to dental services should be part of the institutional health plan (12). Because Dental and Periodontal Diseases may share inflammatory and behavioral pathways with systemic conditions, oral health interventions may also support broader chronic disease prevention (32, 33). This perspective is increasingly reflected in Western health systems, where oral health is more commonly integrated into preventive care (34, 35). In the United States, approximately 42% of adults aged 30 years or older have periodontitis (36), and 65% reported receiving a dental examination or professional cleaning within the previous year (37). European epidemiological surveys have similarly shown a high periodontal burden while emphasizing routine preventive maintenance as part of community health programs (12, 38). Together with our findings, these data suggest that oral health in healthcare workers should not be viewed as an isolated dental issue, but rather as a component of long-term occupational chronic disease prevention.

Several limitations should be acknowledged. First, the analysis was based on aggregated annual institutional reports rather than individual-level longitudinal records. Because the same employee may have contributed data across multiple years, incidence, persistence, remission, and within-person changes could not be evaluated. Second, the aggregated design precluded multivariable adjustment, and the observed temporal patterns may have been influenced by residual confounding from age structure, workforce composition, department, occupation, shift work, health behaviors, medication use, or other unmeasured factors. In addition, although lifestyle information was available in annual reports, these variables were not consistently incorporated into the standardized multi-year statistical tables and therefore could not be formally linked to abnormality prevalence. Third, the analyzed outcomes primarily represented detected health examination abnormalities identified through screening procedures rather than clinically confirmed diagnoses established through comprehensive diagnostic evaluation or longitudinal follow-up. Several findings, particularly Thyroid Nodules, Breast Nodules, ultrasound-suspected Hashimoto Thyroiditis, and ultrasound-detected Hepatic Steatosis, may therefore reflect screening-detected abnormalities requiring further clinical assessment rather than definitive disease status. In addition, Dyslipidemia and Hyperuricemia were defined according to laboratory abnormalities identified during routine health examinations rather than confirmed chronic disease diagnoses. Some abnormalities, particularly Thyroid Nodules and Breast Nodules, are also inherently influenced by screening intensity, ultrasound resolution, and reporting practices. Therefore, increased detection may not necessarily reflect a parallel increase in clinically significant disease. Finally, age-stratified abnormality data were not consistently available across all four annual reports, precluding analyses of age-specific prevalence patterns and temporal trends.

Despite these limitations, this study has several strengths. It covered four consecutive annual examination cycles, included more than 14,000 annual examination records, and maintained consistently high participation rates. Sex-stratified analyses identified distinct health risk patterns that would have been obscured in overall prevalence estimates. In addition, trend analyses provided a quantitative summary of temporal changes and helped identify priority conditions for targeted intervention. Because the data were derived from an ongoing hospital employee health examination program, the findings have direct relevance for occupational health planning and institutional health management.

The findings also have practical implications. For male employees, priority should be given to integrated cardiometabolic prevention focusing on body weight, lipid abnormalities, uric acid, and Hepatic Steatosis, supported by annual risk stratification, individualized feedback, exercise promotion, dietary intervention, and follow-up for employees with multiple abnormalities. For female employees, thyroid and breast screening should be linked to standardized imaging classification and structured referral for higher-risk findings. Across the entire workforce, oral health should be recognized as a core component of occupational health management rather than a minor ancillary finding. At the system level, future health examinations would benefit from the establishment of a de-identified individual-level longitudinal database incorporating demographic characteristics, occupational factors, lifestyle variables, laboratory findings, imaging classifications, medication exposure, and follow-up outcomes.

In conclusion, annual staff health examinations at Shanxi Bethune Hospital from 2022 to 2025 showed high examination uptake and a substantial burden of oral, metabolic, endocrine, and sex-specific abnormalities. Male employees had markedly higher prevalence of Dyslipidemia, Hepatic Steatosis, Hyperuricemia, and Overweight or Obesity, whereas female employees had higher prevalence of Thyroid Nodules, Hashimoto Thyroiditis, and Breast Nodules. Several abnormalities increased over time, particularly Thyroid Nodules, Hepatic Steatosis, Hashimoto Thyroiditis, Overweight or Obesity, and Breast Nodules. These findings support a targeted employee health management strategy integrating cardiometabolic prevention, oral health services, thyroid and breast follow-up pathways, and improved longitudinal health data infrastructure.

## Data Availability

The original contributions presented in the study are included in the article/Supplementary material, further inquiries can be directed to the corresponding authors.

## References

[1] Khosravipour M Khanlari P Khazaie S Khosravipour H Khazaie H. A systematic review and meta-analysis of the association between shift work and metabolic syndrome: the roles of sleep, gender, and type of shift work. Sleep Med Rev. (2021) 57:101427. doi: 10.1016/j.smrv.2021.101427, 33556868

[2] Lu Y Zhang H Lu J Ding Q Li X Wang X . Prevalence of Dyslipidemia and availability of lipid-lowering medications among primary health care settings in China. JAMA Netw Open. (2021) 4:e2127573. doi: 10.1001/jamanetworkopen.2021.27573, 34586366 PMC8482054

[3] Pan XF Wang L Pan A. Epidemiology and determinants of obesity in China. Lancet Diabetes Endocrinol. (2021) 9:373–92. doi: 10.1016/S2213-8587(21)00045-0, 34022156

[4] Younossi ZM Golabi P Paik JM Henry A Van Dongen C Henry L. The global epidemiology of nonalcoholic fatty liver disease (NAFLD) and nonalcoholic steatohepatitis (NASH): a systematic review. Hepatology. (2023) 77:1335–47. doi: 10.1097/HEP.0000000000000004, 36626630 PMC10026948

[5] Li Y Shen Z Zhu B Zhang H Zhang X Ding X. Demographic, regional and temporal trends of hyperuricemia epidemics in mainland China from 2000 to 2019: a systematic review and meta-analysis. Glob Health Action. (2021) 14:1874652. doi: 10.1080/16549716.2021.1874652, 33475474 PMC7833047

[6] Islam MS Wei P Suzauddula M Nime I Feroz F Acharjee M . The interplay of factors in metabolic syndrome: understanding its roots and complexity. Mol Med. (2024) 30:279. doi: 10.1186/s10020-024-01019-y, 39731011 PMC11673706

[7] Fahed G Aoun L Bou Zerdan M Allam S Bou Zerdan M Bouferraa Y . Metabolic syndrome: updates on pathophysiology and management in 2021. Int J Mol Sci. (2022) 23. doi: 10.3390/ijms23020786, 35054972 PMC8775991

[8] Gutierrez-Cuevas J Santos A Armendariz-Borunda J. Pathophysiological molecular mechanisms of obesity: a link between MAFLD and NASH with cardiovascular diseases. Int J Mol Sci. (2021) 22. doi: 10.3390/ijms222111629, 34769060 PMC8583943

[9] Li Y Jin C Li J Tong M Wang M Huang J . Prevalence of thyroid nodules in China: a health examination cohort-based study. Front Endocrinol (Lausanne). (2021) 12:676144. doi: 10.3389/fendo.2021.676144, 34122350 PMC8188053

[10] Mu C Ming X Tian Y Liu Y Yao M Ni Y . Mapping global epidemiology of thyroid nodules among general population: a systematic review and meta-analysis. Front Oncol. (2022) 12:1029926. doi: 10.3389/fonc.2022.1029926, 36439513 PMC9685339

[11] Bray F Laversanne M Sung H Ferlay J Siegel RL Soerjomataram I . Global cancer statistics 2022: GLOBOCAN estimates of incidence and mortality worldwide for 36 cancers in 185 countries. CA Cancer J Clin. (2024) 74:229–63. doi: 10.3322/caac.21834, 38572751

[12] Nascimento GG Alves-Costa S Romandini M. Burden of severe periodontitis and edentulism in 2021, with projections up to 2050: the global burden of disease 2021 study. J Periodontal Res. (2024) 59:823–67. doi: 10.1111/jre.13337, 39192495

[13] Von Elm E Altman DG Egger M Pocock SJ Gøtzsche PC Vandenbroucke JP. The strengthening the reporting of observational studies in epidemiology (STROBE) statement: guidelines for reporting observational studies. Lancet. (2007) 370:1453–7. doi: 10.1016/S0140-6736(07)61602-X, 18064739

[14] Foros P Oikonomou E Koletsi D Rahiotis C. Detection methods for early caries diagnosis: a systematic review and Meta-analysis. Caries Res. (2021) 55:247–59. doi: 10.1159/000516084, 34130279

[15] Heitz-Mayfield LJA. Conventional diagnostic criteria for periodontal diseases (plaque-induced gingivitis and periodontitis). Periodontol. (2000) 95:10–9. doi: 10.1111/prd.12579, 38831568

[16] Goldberg JF Hyun G Ness KK Dixon SB Towbin JA Rhea IB . Dyslipidemia and cardiovascular disease among childhood cancer survivors: a St. Jude lifetime cohort report. J Natl Cancer Inst. (2024) 116:408–20. doi: 10.1093/jnci/djad222, 37952244 PMC10919333

[17] Patel RS Pasea L Soran H Downie P Jones R Hingorani AD . Elevated plasma triglyceride concentration and risk of adverse clinical outcomes in 1.5 million people: a CALIBER linked electronic health record study. Cardiovasc Diabetol. (2022) 21. doi: 10.1186/s12933-022-01525-5, 35681241 PMC9185961

[18] Jeon SK Lee JM Joo I Park SJ. Quantitative ultrasound radiofrequency data analysis for the assessment of hepatic steatosis in nonalcoholic fatty liver disease using magnetic resonance imaging proton density fat fraction as the reference standard. Korean J Radiol. (2021) 22:1077–86. doi: 10.3348/kjr.2020.1262, 33739636 PMC8236371

[19] Wu Q Wang Y Chen P Wei J Lv H Wang S . Increased incidence of Hashimoto thyroiditis in selenium deficiency: a prospective 6-year cohort study. J Clin Endocrinol Metab. (2022) 107:e3603–11. doi: 10.1210/clinem/dgac410, 35789269

[20] Zhang Q Zhang S Pan Y Sun L Li J Qiao Y . Deep learning to diagnose Hashimoto's thyroiditis from sonographic images. Nat Commun. (2022) 13:3759. doi: 10.1038/s41467-022-31449-3, 35768466 PMC9243092

[21] Zhao H Lu Z Lu Y. The potential of probiotics in the amelioration of hyperuricemia. Food Funct. (2022) 13:2394–414. doi: 10.1039/d1fo03206b, 35156670

[22] Haam JH Kim BT Kim EM Kwon H Kang JH Park JH . Diagnosis of obesity: 2022 update of clinical practice guidelines for obesity by the Korean Society for the Study of obesity. J Obes Metab Syndr. (2023) 32:121–9. doi: 10.7570/jomes23031, 37386771 PMC10327686

[23] Russ G Bonnema SJ Erdogan MF Durante C Ngu R Leenhardt L. European thyroid association guidelines for ultrasound malignancy risk stratification of thyroid nodules in adults: the EU-TIRADS. Eur Thyroid J. (2017) 6:225–37. doi: 10.1159/000478927, 29167761 PMC5652895

[24] Fleury E Marcomini K. Performance of machine learning software to classify breast lesions using BI-RADS radiomic features on ultrasound images. Eur Radiol Exp. (2019) 3:34. doi: 10.1186/s41747-019-0112-7, 31385114 PMC6682836

[25] Strack C Behrens G Sag S Mohr M Zeller J Lahmann C . Gender differences in cardiometabolic health and disease in a cross-sectional observational obesity study. Biol Sex Differ. (2022) 13:8. doi: 10.1186/s13293-022-00416-4, 35246259 PMC8897897

[26] Kammerlander AA Lyass A Mahoney TF Massaro JM Long MT Vasan RS . Sex differences in the associations of visceral adipose tissue and cardiometabolic and cardiovascular disease risk: the Framingham heart study. J Am Heart Assoc. (2021) 10:e019968. doi: 10.1161/JAHA.120.019968, 33998254 PMC8483556

[27] Muscogiuri G Verde L Vetrani C Barrea L Savastano S Colao A. Obesity: a gender-view. J Endocrinol Investig. (2024) 47:299–306. doi: 10.1007/s40618-023-02196-z, 37740888 PMC10859324

[28] Cooper AJ Gupta SR Moustafa AF Chao AM. Sex/gender differences in obesity prevalence, comorbidities, and treatment. Curr Obes Rep. (2021) 10:458–66. doi: 10.1007/s13679-021-00453-x, 34599745

[29] Xu L Zeng F Wang Y Bai Y Shan X Kong L. Prevalence and associated metabolic factors for thyroid nodules: a cross-sectional study in southwest of China with more than 120 thousand populations. BMC Endocr Disord. (2021) 21:175. doi: 10.1186/s12902-021-00842-2, 34452638 PMC8400405

[30] Ospina NS Papaleontiou M. Thyroid nodule evaluation and Management in Older Adults: a review of practical considerations for clinical endocrinologists. Endocr Pract. (2021) 27:261–8. doi: 10.1016/j.eprac.2021.02.003, 33588062 PMC8092332

[31] Hu X Chen Y Shen Y Tian R Sheng Y Que H. Global prevalence and epidemiological trends of Hashimoto's thyroiditis in adults: a systematic review and meta-analysis. Front Public Health. (2022) 10:1020709. doi: 10.3389/fpubh.2022.1020709, 36311599 PMC9608544

[32] Hajishengallis G Chavakis T. Local and systemic mechanisms linking periodontal disease and inflammatory comorbidities. Nat Rev Immunol. (2021) 21:426–40. doi: 10.1038/s41577-020-00488-6, 33510490 PMC7841384

[33] Bhuyan R Bhuyan SK Mohanty JN Das S Juliana N Juliana IF. Periodontitis and its inflammatory changes linked to various systemic diseases: a review of its underlying mechanisms. Biomedicine. (2022) 10. doi: 10.3390/biomedicines10102659, 36289921 PMC9599402

[34] Fellows JL Atchison KA Chaffin J Chavez EM Tinanoff N. Oral health in America: implications for dental practice. J Am Dent Assoc. (2022) 153:601–9. doi: 10.1016/j.adaj.2022.04.002, 35643534 PMC9637008

[35] Patel J Wallace J Doshi M Gadanya M Ben Yahya I Roseman J . Oral health for healthy ageing. Lancet Healthy Longev. (2021) 2:e521–7. doi: 10.1016/S2666-7568(21)00142-2, 36098001

[36] Hou K Song W He J Ma Z. The association between non-high-density lipoprotein cholesterol to high-density lipoprotein cholesterol ratio (NHHR) and prevalence of periodontitis among US adults: a cross-sectional NHANES study. Sci Rep. (2024) 14:5558. doi: 10.1038/s41598-024-56276-y, 38448487 PMC10918089

[37] Ren Z Xue Y Zhang H Zhang T Wang M Guo T . Association between probiotic consumption and periodontitis: evidence from NHANES 2009-2014. J Clin Periodontol. (2023) 50:1476–86. doi: 10.1111/jcpe.13865, 37596796

[38] Trindade D Carvalho R Machado V Chambrone L Mendes JJ Botelho J. Prevalence of periodontitis in dentate people between 2011 and 2020: a systematic review and meta-analysis of epidemiological studies. J Clin Periodontol. (2023) 50:604–26. doi: 10.1111/jcpe.13769, 36631982

