# Supplementary material for: Four-year trends in health findings among hospital staff an ecological time-series study 2022–2025
Source: Front Public Health. 2026 Oct 7;14:1890585. doi: 10.3389/fpubh.2026.1890585 (PMC13683792; doi:10.3389/fpubh.2026.1890585)
Supplement: Supplementary file 1 [file Table_1.docx]

## sTable 1. Annual High-Risk Breast Imaging Findings

| Year | BI-RADS 4a | BI-RADS 4b | BI-RADS 4c | Total BI-RADS ≥4 | Follow-up breast cancer |
| --- | --- | --- | --- | --- | --- |
| 2022 | 4 | 0 | 0 | 4 | 0 |
| 2023 | 7 | 0 | 0 | 7 | 0 |
| 2024 | 6 | 1 | 0 | 7 | 2 |
| 2025 | 6 | 1 | 1 | 8 | 2 |
